# Iatrogenic right coronary artery dissection distal to a total occlusion: a case report

**DOI:** 10.4076/1757-1626-2-6797

**Published:** 2009-07-02

**Authors:** Demetrios Antoniades, Stavros Apostolakis, Spiros Tzoras, Kyriakos Lazaridis

**Affiliations:** Department of Cardiology, Army Veterans HospitalAthensGreece

## Abstract

**Introduction:**

Coronary artery dissections with or without rupture is a rare but well-recognized complication of coronary angiography with a high morbidity and mortality rate.

**Case presentation:**

We present a rare case of right coronary artery dissection distal to a totally occluded vessel. The vessel dissected during the second injection of contrast agent without any direct mechanical manipulation (catheter or guide-wire induced). Hopefully the dissection had no clinical consequences and the patient was discharged after 48 hour intensive monitoring.

**Conclusions:**

We believe that the contrast agent that was forced in the proximal part of the RCA increased through the anastomotic branches the sheer stress on the diseased endothelium of the distal artery causing it to dissect. It is an instructive -not previously described- phenomenon that underscores that atherosclerotic tissue is unpredictable and should be treated with extreme caution.

## Introduction

Extensive coronary artery dissections with or without rupture occasionally occur during percutaneous interventions [1-3]. It is more commonly in the right than in the left coronary artery probably due to histological differences in the proximal vessel [4]. Dissection is usually guide-catheter, guide-wire or balloon inflation induced, and it further propagates by contrast injection and/or coronary flow [1-4]. Conservative treatment may be adequate for limited dissections. Extensive dissections with pericardial extravasation require immediate treatment. Although emergency coronary artery by pass grafting (CABG) is effective, it is a time consuming procedure that entails the risk of irreversible myocardial damage. The use of covered stents has obviated the need of high risk emergency surgery [2,4].

## Case presentation

We report the case of a 72-year-old Caucasian male, referred to our department for further investigation of recent-onset effort angina. He was a non-smoker, hypertensive and dyslipidemic patient under treatment with ACE inhibitor and statin. No parental history of cardiovascular disease was reported to us.

On admission he was free of symptoms at rest and he had a relatively normal clinical examination. He also had a normal chest X-ray, and laboratory tests yielded no unusual findings besides alter lipoprotein levels (predominantly low HDL).

The patient was subjected to SPECT myocardial perfusion imaging before and after Bruce protocol graded exercise stress test. During the second stage of exercise stress test the patient reported mild chest discomfort and he gradually developed down-sloping ST depression of approximately 1.5 mm at the inferior leads. Exercise was halted at 6:02 due to patient’s fatigue.

SPECT myocardial perfusion imaging revealed an exercise-induced significant inferio-lateral perfusion defect.

The patient was then subjected to coronary angiography (CA), which yielded total occlusion of right coronary artery proximally. The distal part of the artery was well perfused by a wide network of anastomotic branches interconnected to the proximal part of RCA. A lesion in the proximal left anterior descending artery (LAD) was also noted. The lesion compromised the 50% of the arterial lumen diameter. The ejection fraction was normal. The patient was put on aspirin, beta-blocker and calcium channel antagonist and he was discharged 24 hours later. On regular follow-up he was in good condition and did not report any symptoms.

**Figure 1.**
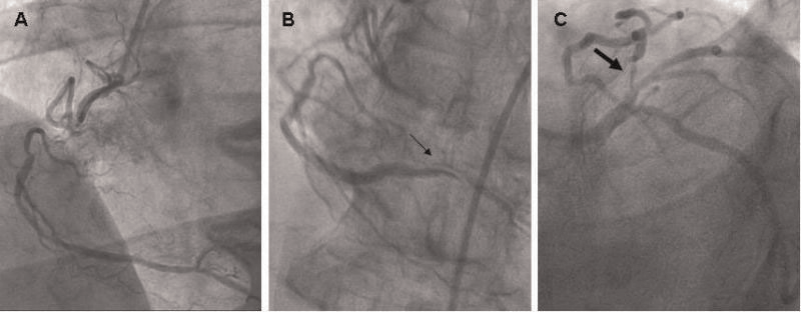
Coronary angiography yielded total occlusion of right coronary artery proximally. The distal part of the artery was perfused by a wide network of anastomotic branches interconnected to the proximal part of right coronary artery **(A)**. The second attempt to visualize the right coronary artery resulted in dissection of the vessel distal to the total occlusion **(B)**. A lesion in the proximal left anterior descending artery was also noted. The lesion compromised the 90% of the arterial lumen diameter **(C)**.

Eight months later and while still on optimal anti-aginal treatment the patient reported relapse of effort angina. He was admitted to our department and he was re-subjected to a CA. The severity of the LAD lesion was subsequently worsened (Figure 1C), while the anatomy of RCA was unchanged (Figure 1A). However on the second attempt to visualize the RCA from the left anterior oblique view, the vessel dissected distally to the total occlusion (Figure 1B and Video 1). Hopefully, the patient remained symptom-free, and hemodynamically stable. Moreover, since the dissection occurred distal to a totally occluded artery, any percutaneous intervention in the dissected lesion was technically impossible. Therefore, the patient was admitted in the intensive care unit and was discharged after 48 hours monitoring. He was referred to the cardiothoracic surgery department for further management of the LAD stenosis which was considered as the symptom-related lesion.

## Discussion

Coronary angiography has become a routine diagnostic procedure in current clinical practice. Complications are uncommon, but still occur during CA and in some instances they can be life-threatening. Catheter-induced dissection of a coronary artery is a rare but well-recognized complication of CA with a high mortality rate if it is left untreated [5,6].

The exact mechanism of iatrogenic coronary artery dissection (ICAD) is not clear. It has been related to vigorous hand-injection of contrast medium, subintimal passage of the guide-wire, or inappropriate handling of the guide-wire catheter [4]. In all instances mechanical straining and shearing forces during CA result in increased wall stress. The atherosclerotic-dysfunctional relatively rigid arterial wall is prone to rapture and can easily dissect if not treated with care [1-5].

Nevertheless, in our case no direct manipulation of the distal part of the vessel could have been performed since it was proximally occluded and no guide-wire was inserted distally to the occlusion. It is however plausible that the contrast agent that was forced in the proximal part of the RCA increased through the anastomotic branches the sheer stress on the diseased endothelium of the distal artery causing it to dissect. Hopefully, the clinical stability of the patient made unnecessary any intervention in the dissected vessel. However the outcome might not have been favorable if the dissection had been complicated with rapture. It would be technically challenging if not impossible the placement of a covered stent at the dissected vessel while surgical treatment of the dissection-a more technically feasible alternative- would have been time consuming.

In conclusion, we report a case of iatrogenic coronary artery dissection distal to a totally occluded RCA. Such a complication has never been reported before and it was rather instructive. It puts forward that injection of contrast medium should be performed with extreme caution especially in patients with severe coronary artery disease. It also reminds that direct mechanical manipulation is not the only way to cause coronary artery dissection.

## Supplementary file

Video 1. The video (MP4 – 4.2MB) can be accessed here: http://casesjournal.com/casesjournal/article/downloadSuppFile/6797/19302

## Abbreviations

ACE: angiotensin-converting enzyme
CA: coronary angiography
CABG: coronary artery bypass graft
HDL: high density lipoprotein
ICAD: iatrogenic coronary artery dissection
LAD: left anterior descending
RCA: right coronary artery
SPECT: single photon emission computed tomography
